# Endothelial Cell Organization Drives Distinct Agonist‐Specific Ca^2+^ Dynamics in Arteries and Veins

**DOI:** 10.1111/apha.70132

**Published:** 2025-11-17

**Authors:** M. D. Lee, R. A. Clark, C. Buckley, X. Zhang, P. Uhlen, C. Wilson, J. G. McCarron

**Affiliations:** ^1^ Strathclyde Institute of Pharmacy and Biomedical Sciences University of Strathclyde Glasgow UK; ^2^ Electronic and Electrical Engineering University of Strathclyde Glasgow UK; ^3^ Department of Medical Biochemistry and Biophysics Karolinska Institutet Stockholm Sweden

**Keywords:** arteries, calcium imaging, endothelial signaling, hub cells, network analysis, veins

## Abstract

**Aim:**

The endothelium regulates cardiovascular function by detecting and interpreting multiple extracellular signals from blood and surrounding tissues, even when these inputs are complex and conflicting. The major challenge faced by the endothelium is decoding this dynamic chemical environment to produce coordinated endothelial cellular responses. In addition to the problems of detection, extracellular signals must be processed correctly intracellularly to generate a functional outcome.

**Methods:**

Ca^2+^ imaging, network analysis and spectral graph theory across ~1000 endothelial cells in intact arteries and veins.

**Results:**

The venous endothelial cell population forms distinct, non‐overlapping communities, each tuned to specific agonists. Within these communities, responsive cells act as bridges, linking members through the most direct communication route. Activation of one cell increases the likelihood of activation occurring in its neighbors, creating localized zones of high responsiveness. Only a small (5%) subset of cells responds to multiple activators. These multifunctional cells form unique connections that integrate and distribute signals between the agonist‐specific sensing communities. We also show that different agonists elicit unique signaling patterns determined by the stimulus, not by intrinsic cellular properties. Finally, signal decoding strategies differ across vascular beds: venous endothelial cells rely on Ca^2+^ signal frequency, while arterial cells use signal amplitude.

**Conclusion:**

The endothelium comprises functionally specialized populations. A small subset of pharmacologically distinct cells plays a key role in signal integration. These hubs are especially vulnerable to disconnection and dysfunction in disease, highlighting them as potential therapeutic targets. The findings presented reveal specialized encoding strategies that distinguish the arterio–venous axis.

## 1 Introduction

The endothelial cell lining of arteries and veins regulates blood flow, pressure, and immune responses. In arteries, the mechanisms by which endothelial cells regulate vascular function have been extensively characterized. However, corresponding regulatory mechanisms in venous endothelial cells (VECs) are poorly understood. Addressing this knowledge gap is important because veins are primary sites for the development of cardiovascular and inflammatory disease with venous endothelial dysfunction contributing to hypertension, chronic inflammation, and sepsis [1, 2].

Regardless of anatomical location (arterial vs. venous), endothelial cells are continuously exposed to circulating factors, mechanical forces, and signals from adjacent tissues and cells. All must be integrated to generate appropriate vascular responses [3]. Indeed, the endothelium processes complex signal arrays from hormones, neurotransmitters, pericytes, smooth muscle cells, various blood cells, viral or bacterial infection and proinflammatory cytokines. The integration requires sensitive and selective detection of individual signals and signal combinations. To then generate coordinated vascular responses from this complex signal processing, endothelial cells must synchronize their own activities across the vessel wall.

Despite widespread acceptance that endothelial cells are heterogeneous [4, 5, 6, 7], functional studies still typically assume coordinated responses emerge through uniform cellular contributions. This assumption treats the endothelium as a collection of equivalent units that respond proportionally to stimuli and contribute equally to vascular control. However, this perspective overlooks the functional implications of cellular diversity. Endothelial cells within individual vessel segments exhibit distinct response profiles to the same stimuli, with specific subpopulations showing preferential activation patterns [8, 9]. This selective responsiveness creates spatially organized functional domains that enable parallel signal processing rather than synchronized uniform activation [10, 11, 12, 13, 14]. Such organization allows simultaneous detection and processing of multiple stimuli through specialized cellular networks, revealing complexity far exceeding uniform activation models and fundamentally altering how we understand endothelial coordination. However, this functional heterogeneity raises questions about how specialized cell populations achieve synchronized vascular control and how they might be targeted therapeutically in disease.

If endothelial signaling depends on specialized subpopulations, rather than uniform responses, then coordination of activities must rely on a subset of strategically positioned cells that integrate and distribute information across the network. Understanding endothelial cell coordination therefore requires the identification of key cells that serve as communication hubs within the overall cell network. These critical hub cells can be identified based on their connections, their influence, or their role in linking other cells. For example, a cell's importance may be determined by the number of neighbors it has, its position at a critical junction or bottleneck for information flow, or its overall connectivity to other influential cells within the endothelial network.

Identifying hub cells is only the first step. Equally important is understanding the language the cells use to communicate. In the endothelium, that language is Ca^2+^ signaling, the universal currency through which cells both process incoming cues and broadcast information across the network. Ca^2+^ signaling is activated by most physiological and pathological stimuli in both venous and arterial endothelium [15]. Changes in intracellular Ca^2+^ mediate diverse cellular processes, including the release of vasoactive factors that regulate vessel tone and structural changes that control vascular permeability and leukocyte transmigration [7, 16]. Ca^2+^ signaling also enables intercellular communication across the endothelial network. Given the role of Ca^2+^ in both intracellular signaling and network coordination, understanding how endothelial cells decode and transmit Ca^2+^ signals is essential for determining how these networks achieve coordinated cardiovascular control.

Ca^2+^ communication networks have been widely studied in intact artery endothelium. However, few studies have examined any component of Ca^2+^ responses in the intact venous endothelium [17, 18, 19]. This is largely due to technical challenges associated with studying the delicate and thin‐walled structure of small veins. Consequently, most investigations into venous endothelial Ca^2+^ have been conducted using cultured cells. While cultured cells offer a controlled experimental condition, it is important to note that endothelial cell phenotype is regulated by local environmental factors, and their functional characteristics can change rapidly in culture [20, 21]. A fundamental unresolved question regarding venous endothelial Ca^2+^ signaling is whether it utilizes mechanisms similar to those described in arteries.

In this study, we used network analysis combined with spectral graph theory and centrality measures to quantify spatial coordination of endothelial cell responses within intact vessel cell populations. By examining heterogeneous Ca^2+^ signaling patterns in both venous and arterial systems, we defined the spatial organization and communication dynamics that define distinct physiological roles in both vascular compartments.

Our findings reveal that venous endothelial cells organize into distinct, signal‐specific functional groups that coordinate stimulus responses and enable simultaneous multi‐signal (parallel) processing. We demonstrate that a subset of cells respond to multiple stimuli and occupy strategic positions within the venous endothelial cell network that facilitates communication between agonist‐specific communities. Within signal‐specific groups, neighboring cell activity influences individual Ca^2+^ event probability without altering intrinsic event characteristics. This finding highlights the importance of intercellular communication in facilitating synchronized responses among VECs while preserving the signaling integrity of each individual cell.

Furthermore, our study demonstrates that venous endothelial cells primarily use frequency modulation to transmit information intracellularly and regulate vasodilation, while arterial endothelial responses are predominantly determined by Ca^2+^ signal amplitude. This difference in encoding mechanisms highlights the divergent Ca^2+^ responses between different vessels of the cardiovascular system.

## 2 Methods

### 2.1 Animals

All animal care and experimental procedures complied with the relevant UK Home Office Regulations, (Schedule 1 of the Animals [Scientific Procedures] Act 1986, UK; License X56B4FB08) and were approved by the University of Strathclyde Animal Welfare and Ethical Review Body. Male Sprague–Dawley rats (10–12 week‐old; 250–300 g), from an in‐house colony, were used for the study and euthanised by cervical dislocation. All material submitted conforms with good publishing practice in physiology [22].

### 2.2 Imaging of Endothelial Ca^2+^ Responses

Immediately following euthanasia, the mesenteric bed was removed and placed in a physiological saline solution (PSS). Second or third‐order mesenteric veins or arteries were cleaned of connective tissue and fat. The vessels were cut open longitudinally and pinned, endothelial side up, on a Sylgard block. The endothelium was preferentially loaded with the fluorescent Ca^2+^ indicator, Cal‐520/AM (5 μM with 0.02% Pluronic F127 and 0.2% DMSO in PSS) at 37°C for 30 min. Following incubation, the vessels were gently washed with PSS and mounted in a custom chamber designed for use on an inverted microscope. Endothelial Ca^2+^ imaging was performed at 10 Hz using an inverted fluorescence microscope (TE2000U, Nikon, Tokyo, Japan) with a 40× objective (1.3 NA; Nikon, Tokyo, Japan) and an EMCCD camera (iXon 888; Andor, Belfast, UK). Cal‐520/AM was excited with 488 nm widefield epifluorescence illumination provided by an LED (CoolLED, United Kingdom).

Following a 5‐min wash with PSS, blood vessels were allowed to equilibrate (30‐min) before the addition of pharmacological agents. In experiments investigating the effects of pharmacological intervention, drugs were flowed into the chamber for 5 min, incubated for 20 min, and remained present throughout recordings. In concentration‐response experiments, full agonist concentration‐response curves were constructed on a single field of endothelium from a single blood vessel. Non‐cumulative concentration‐response curves were generated by sequentially superfusing blood vessels with increasing agonist concentrations, followed by a 5‐min wash with PSS and a 5‐min re‐equilibration between each concentration.

### 2.3 Ca^2+^ Event Detection and Analysis

**FIGURE 1:**
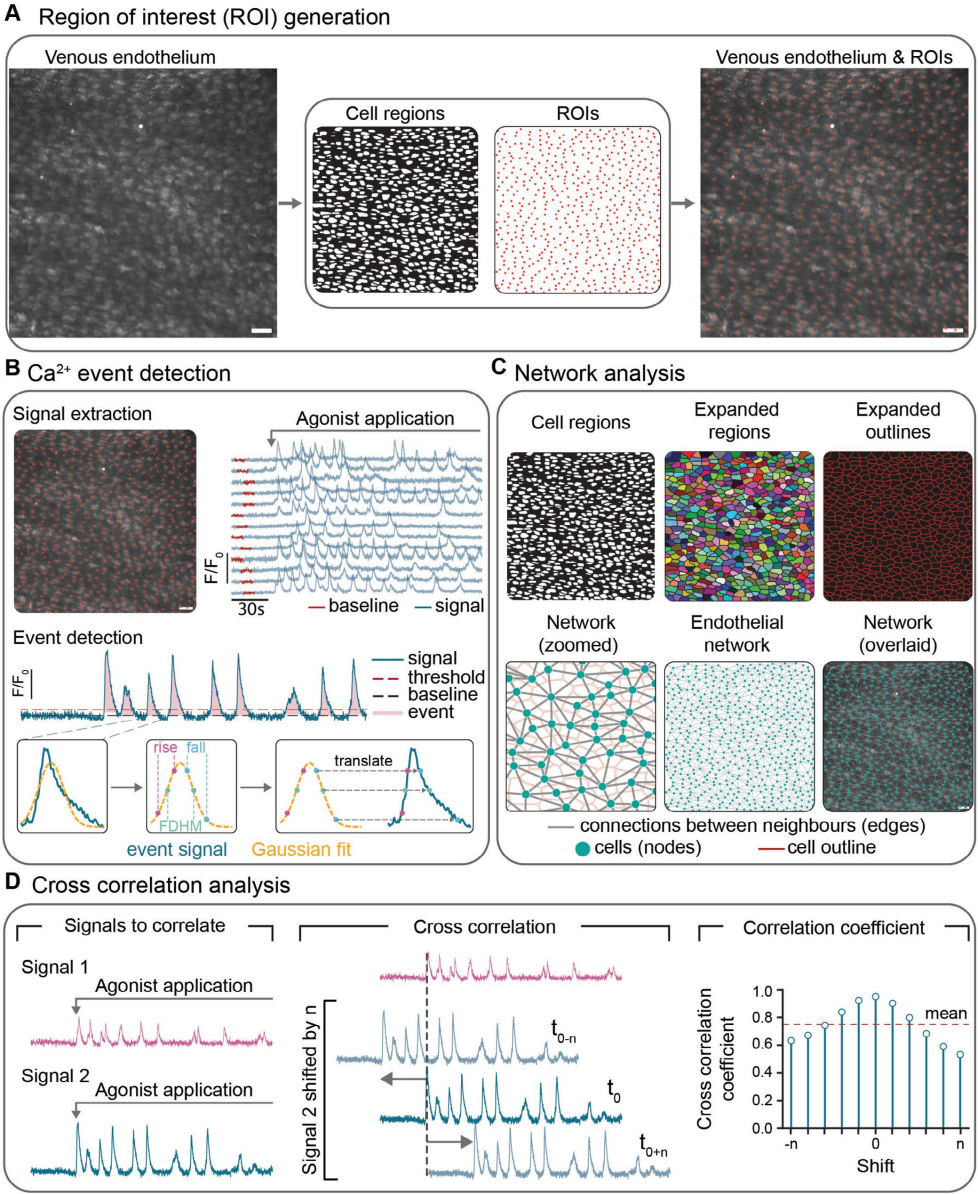
Analysis of endothelial cell Ca^2+^ responses. (A) Representative image showing ∼500 endothelial cells from an *en face* second‐order mesenteric vein (left). Regions of interest (ROIs) are identified within the cell regions (middle) using the Python library, Napari, and overlaid on the venous endothelium (right). (B) Ca^2+^ signals (F/F₀) were extracted from all ROIs. 13 representative signals are shown. The red‐highlighted section of each trace denotes the baseline region identified by the software. A Gaussian function was fitted to each detected event (Ca^2+^ rise above threshold), and rise time, fall time, and full duration at half maximum (FDHM) were calculated. These parameters were translated to the real event signal to obtain values for each Ca^2+^ transient. (C) Cell regions were expanded until they contacted neighboring cells. Cells that touched were classified as neighbors, and connections (edges) were drawn between adjacent cells (nodes) to form a structural network. The reconstructed endothelial network is overlaid on the venous endothelium. (D) Cross‐correlation analysis between two Ca^2+^ signals. One signal (signal 2) is shifted relative to the other (signal 1), and the correlation coefficient is calculated for each time shift. Scale bars, 50 μm.

Agonist‐evoked Ca^2+^ responses were analyzed using custom analysis software written in the Python programming language as previously described [23, 24]. In brief, regions of interest (ROIs) were generated for each endothelial cell as described previously [11, 25] and fluorescence signals from each ROI were extracted and smoothed using a Savitzky‐Golay (21 point, third‐order) filter, and expressed as fractional changes in fluorescence (F/F_0_) from baseline (Figure 1A,B). The baseline was automatically detected (as the 10‐s region with the lowest activity) before agonist application (Figure 1B). Ca^2+^ events were taken as signals that exceeded a threshold of 5 times the standard deviation of baseline fluctuation values (Figure 1B). This threshold was chosen as being large enough to exclude noise fluctuations and changes in fluorescence that occurred due to movement from the analysis, but small enough to detect low‐amplitude Ca^2+^ events. Following event extraction, a least‐squares optimization algorithm was used to fit an exponentially modified Gaussian function to each Ca^2+^ peak to measure event parameters (event frequency, amplitude, rise time, fall time and full duration at half‐maximum [FDHM; Figure 1B]).

### 2.4 Pressure Myography

The reactivity of arteries and veins was assessed using a pressure myograph. Following dissection and removal of surrounding fat and connective tissue, vessels were mounted onto two glass micropipettes and secured with silk suture thread inside a VasoTracker myograph chamber filled with PSS solution [26]. One end of the glass cannula was connected to a servo‐controlled peristaltic pump for precise pressure application. Pressure was gradually increased in veins (from 2 mmHg to 8 mmHg in 1 mmHg increments) and arteries (from 20 mmHg to 70 mmHg in 10 mmHg increments), with 10‐min equilibration periods between each pressure increment. To ensure uniform tension and avoid vessel buckling, blood vessels were stretched longitudinally.

To confirm vessel integrity, veins were checked for leaks by stopping the flow of PSS from the pressure servo. Any vessel that showed a pressure drop was excluded from the study. PSS was continuously circulated through the chamber at 5 mL/min using a peristaltic pump connected to a reservoir heated to 37°C. Once the final pressure was reached, the vessels were allowed to equilibrate for 30 min.

Following equilibration, vessels were contracted to 80% of their initial diameter using phenylephrine [27]. Concentration‐response curves were generated by cumulatively adding acetylcholine (ACh) or bradykinin at concentrations ranging from 1 nM to 10 μM in half‐log increments to the mounted vessel. Dilation was expressed as a percentage of the phenylephrine‐induced contraction. Images of vessels were recorded using a CMOS camera (DCC1545; ThorLabs Inc. Newton, New Jersey, USA; 5.2 μm pixel size) through a 4× (air; numerical aperture 0.1; Olympus Plan N) objective lens. Images (8‐bit depth) were captured using VasoTracker software [26]. Vascular reactivity was assessed using the VasoTracker analysis software.

### 2.5 Endothelial Cell Network Analysis and Connectivity

To study endothelial cell connectivity and network organization, we created a graph‐based representation of the endothelial network using Python 3.11 and NetworkX. Cell boundaries were defined using the Python library Napari [28]. In a network, nodes are the individual components, and edges are the connections that link these components together [29, 30]. Structurally, in the endothelium, nodes are individual endothelial cells, and edges are connections between neighboring endothelial cells [11].

To determine if endothelial cells were neighbors of one another, cell boundaries were expanded and boundaries that touched were considered neighbors (Figure 1C). A concave hull (alpha shape) was calculated to define the boundary of cells within the field of view. Any cell that extended beyond the concave hull was marked as a boundary cell. Boundary cells were excluded from the analysis to avoid uncertain connectivity. Graphs were used to compare the Ca^2+^ responses of individual cells with the network‐wide response.

### 2.6 Agonist‐Sensitive Cell Clustering

To examine spatial clustering among agonist‐sensitive cells, we mapped cellular responses to each agonist at its EC_25_ concentration. This concentration was selected to balance two critical experimental considerations: first, it reliably activated a sufficient proportion of cells (≥ 20%) to enable robust statistical analysis, whereas lower concentrations (e.g., EC_20_) may produce inconsistent activation levels that fall below 20% of cell activation; secondly, it limited the number of cells that were active due to propagation of the signal and not direct activation by the agonist. To further enhance this, analysis was also limited to the top 20% of the cell population ranked by response frequency. An active cell with no active neighbors was termed to respond in isolation. An active cell with one or more immediate neighbors that were also active, was classified as a community. Network metrics were calculated to quantify the organization and connectivity of the network, including the degree of clustering, which reflects the number of connections each cell formed within a community, and the clustering coefficient, which quantifies the level of interconnectedness among neighboring cells within a community. These metrics were used to evaluate whether or not the observed clustering of nodes within a community was greater than would be expected from a random distribution.

To assess statistical significance, we used a permutation analysis by randomly reassigning Ca^2+^ responses to generate randomized networks. Each randomization had precisely the same number of active cells as the observed network. This randomization of cell activity was repeated 100 times. The network metrics for each random network were calculated and the mean across the 100 permutations was compared with the observed data.

To assess the spatial overlap of VECs responsive to ACh and bradykinin, we identified the most sensitive cells to each agonist (top 20% ranked by response frequency) and compared their distribution patterns.

### 2.7 Spectral Graph Theory and Betweenness Centrality: Analysis of Endothelial Cell Networks in Signal Propagation

Betweenness centrality and eigenvector‐based centralities were used to determine the roles of individual cells in the diffusion of information across the endothelial network, by identifying key cells that mediate signal propagation or that are well‐connected within highly responsive regions. Betweenness centrality identifies cells that act as key intermediaries, bridging communication pathways by lying on the shortest paths between other nodes [31]. In contrast, eigenvector‐based centralities identify nodes with high influence and central positioning at either a global or local scale, reflecting their ability to efficiently transmit and receive signals across the entire network or within their local neighborhood.

The global and local of eigenvector‐based centralities are computed using the eigenvectors (v) of the adjacency matrix (A) [32]. The first left eigenvector $v_{1}$ is associated with the largest eigenvalue *λ*_1_ of A, where $v_{1}A=\lambda_{1}v_{1}$, and is commonly referred to as eigenvector centrality [32]. This first eigenvector reflects each node's capacity to distribute and receive information throughout the whole (global) network. In endothelial cell networks, this value identifies a central hub of well‐connected nodes capable of efficiently communicating with the rest of the network.

To detect local influence, the largest eigengap value is first found to identify prominent community structures in the network [33]. The eigengap is the difference between two consecutive eigenvalues in the matrix, and indicates how well groups or clusters are separated. The ith eigengap is defined as the difference between adjacent eigenvalues, $\lambda_{i}-\lambda_{i+1}$. For i>1, the largest eigengap reveals the most prominent local community structure present in the network, providing insight into a cell's role in local communication that is distinct from its ability to communicate globally across the whole network. To detect cell influence in localized communities, with reference to the largest eigengap, the communities of dynamical influence are found using the eigenvector associated with the largest eigengap, and those preceding it [34]. This approach defines a matrix $\left[v\_1,...,v\_i\right]$ and calculating the $L_{2}$ norm for each node to identify the local community structure around $\lambda_{i}$ and quantify each node's influence within its community more effectively than by using $v_{i}$ alone [34].

We calculated the betweenness and eigenvector centrality for each node in the structural network and mapped the positions of the top 20% of cells exhibiting the highest frequency of Ca^2+^ events in response to ACh or bradykinin. The corresponding centrality values of these cells were derived based on their positions in the network.

To assess statistical significance, a permutation analysis was conducted. To do this, the active cell positions for each agonist were randomly redistributed while maintaining the same number of active cells as occurred in the observed data. Betweenness and eigenvector centrality values were recalculated for these randomized networks. This randomization was repeated 100 times for each dataset and the mean across the 100 permutations was compared with the observed data.

### 2.8 Neighbor Influence on Ca^2+^ Event Probability

To investigate how neighboring cell activity influences Ca^2+^ signaling, we quantified both the probability of event occurrence and event characteristics (peak amplitude, rise time, fall time, FDHM) as a function of active neighbor count. Active neighbors were defined as cells showing Ca^2+^ events within a time window equal to three times the median FDHM of all Ca^2+^ events.

We implemented a case–control approach: (1) *Positive cases*—target cell events preceded by neighbor activity within the time window; (2) *Negative cases*—neighbor activity not followed by target cell response. For negative cases, all neighbor events were chronologically ordered and grouped into non‐overlapping time windows, where each window began with the first neighbor event and extended for the duration of the analysis window (3× median FDHM). Subsequent neighbor events occurring within this window were included in the same group, ensuring that each neighbor was counted only once. Neighbors were subsequently categorized as cluster (same functional cluster as target) or non‐cluster. Analysis was restricted to homogeneous scenarios where all responding neighbors belonged exclusively to one category (i.e., all neighbors in cluster or all neighbors in non‐cluster), excluding mixed scenarios to enable clear statistical comparisons.

A permutation analysis was conducted to assess statistical significance. For this, the timing of events was randomly redistributed across the network while the number of events per cell remained constant. This ensured that intrinsic cellular activity levels were preserved. The approach maintained the original spatial network topology and functional cluster assignments, creating a null model where temporal relationships between neighbors were disrupted while preserving all other network properties.

Cross‐dataset comparisons included only neighbor counts consistently represented across all datasets within each group to prevent sampling bias. This approach allowed us to test whether neighboring cell activity increases the likelihood of a response and to directly compare Ca^2+^ event parameters within the same cell under different neighbor‐activity conditions, thereby determining whether interactions between neighbors and target cells shape the strength of local network effects on individual responses.

### 2.9 Cross‐Correlation Analysis

To quantify the similarity between two Ca^2+^ signals, we performed cross‐correlation analysis, which evaluates the relationship between two signals over time (Figure 1D). Cross‐correlation measures both the strength and timing of correlation by determining how well two signals align when one is shifted relative to the other (Figure 1D).

To capture both baseline and activity components while focusing on physiologically relevant periods, we used an extended analysis window that included 10 s of pre‐activation baseline plus the full activity period. The shortest combined duration (baseline + activity) across all compared datasets was used to ensure equal comparison windows. When comparing datasets with different activation timings, signals were temporally aligned using their respective activity onset times.

We calculated the cross‐correlation coefficient by shifting one signal relative to the other. To account for potential temporal delays between signals, we allowed time shifts of up to ±*t* seconds, where *t* was determined as three times the median FDHM of all Ca^2+^ events across datasets (Figure 1D). This adaptive time lag window accounts for the characteristic timescales of Ca^2+^ signaling in the preparation.

For each signal pair, we divided the traces into two equal time segments to assess whether correlations differed between the early phase (baseline and initial activation) and the late activation phase. For each segment, we computed the normalized, demeaned cross‐correlation across all time shifts within the lag window and took the maximum value as the measure of optimal temporal alignment during that period. Finally, we calculated the median of the two segment correlations as the final cross‐correlation coefficient, providing a robust measure that accounts for both early and late signal dynamics.

The resulting cross‐correlation coefficient ranged from 0 to 1, where 0 indicates no relationship and 1 indicates a perfect positive match between the signals. Higher values suggest stronger similarity in both shape and timing, while lower values indicate weaker similarity.

We assessed two connectivity types: within‐network (nodes activated by the same stimulus) and between‐network (nodes activated by different stimuli). For each node, correlation coefficients were summarized using the 75th percentile to minimize outlier influence while capturing predominant connectivity strength, enabling quantitative comparison of intra‐network coordination and inter‐network coupling patterns.

### 2.10 Drugs and Solutions

PSS consisted of: 145 mM NaCl, 4.7 mM KCl, 2.0 mM 3‐(N‐morpholino)propanesulfonic acid (MOPS), 1.2 mM NaH_2_PO_4_, 5.0 mM glucose, 0.02 mM ethylenediaminetetraacetic acid (EDTA), 1.17 mM MgCl_2_, and 2.0 mM CaCl_2_, adjusted to pH 7.4 with NaOH. All reagents included in the PSS were obtained from Sigma Aldrich (UK).

Cal‐520/AM was obtained from Abcam (UK). Pluronic F‐127 was obtained from Sichem (Germany). Acetylcholine, bradykinin and phenylephrine were obtained from Sigma Aldrich (USA).

### 2.11 Data Presentation and Statistical Analysis

Summarized data are presented as mean ± S.E.M. values; *n* refers to the number of animals. Data were compared using a two‐tailed Student's *t*‐test (paired data), a Welch's *t*‐test (unpaired data) or repeated measures one‐way ANOVA with Dunnett's test for multiple comparisons as appropriate and described in figure captions. Sigmoidal or bell‐shaped curves were fitted to concentration‐response data. Linear regression analysis was performed to assess the relationship between the number of active neighbors and event probability or event characteristics, with goodness of fit reported as r [2] values. All statistical tests were performed using GraphPad Prism, version 6.0 (GraphPad Software). A *p* value of < 0.05 was accepted as statistically significant.

## 3 Results

### 3.1 Venous and Arterial Endothelial Cell Morphology

**FIGURE 2:**
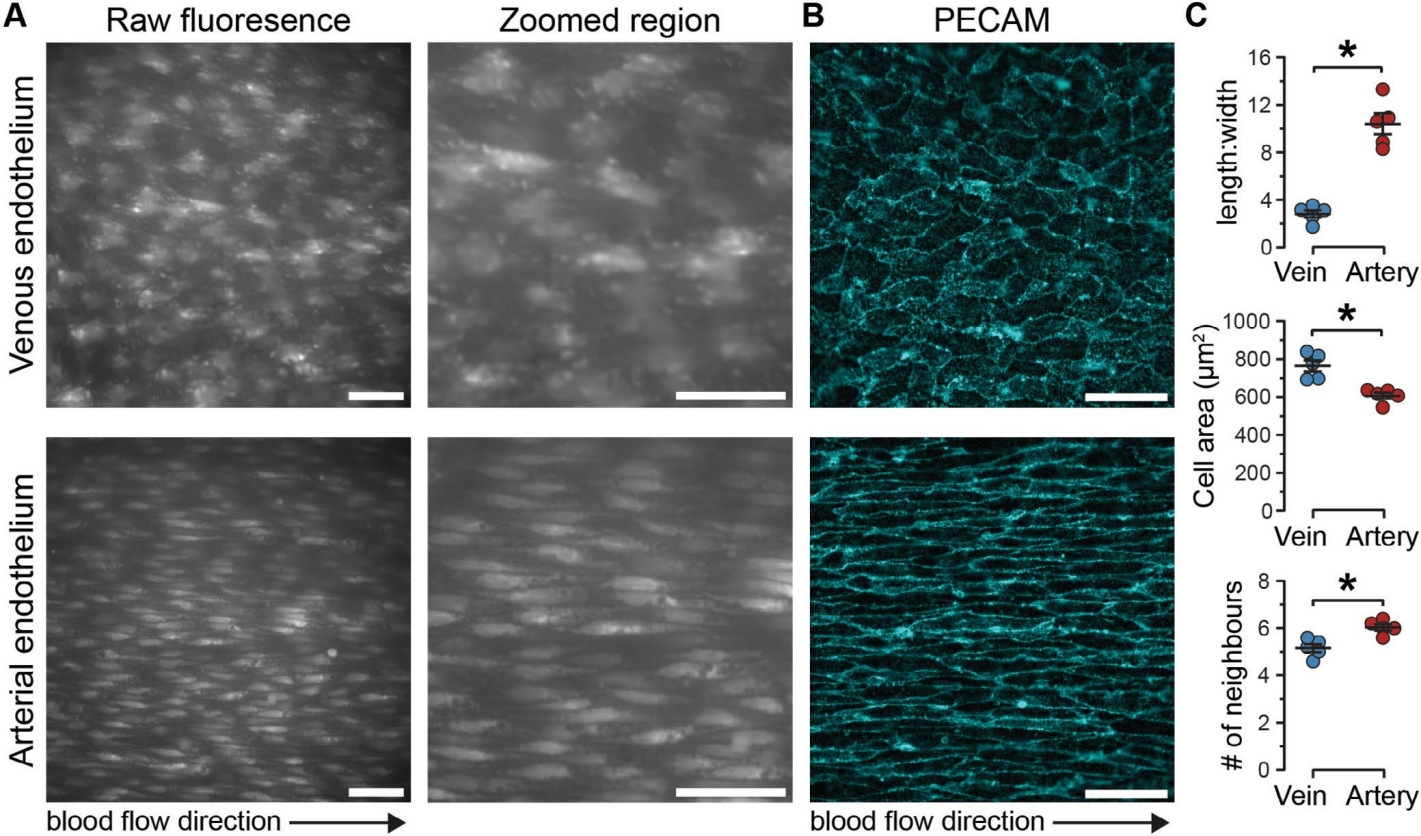
Morphology of venous and arterial endothelial cells in intact blood vessels. (A) Representative image showing endothelial cells from an *en face* second‐order mesenteric vein (top) and second‐order mesenteric artery (bottom) each loaded with a fluorescent Ca^2+^ indicator (Cal‐520/AM; left) and zoomed region (right). (B) Immunostaining against platelet endothelial cell adhesion molecule (PECAM). (C) Summary data of endothelial cell connectivity and morphology, *n* = the number of animals. Each replicate is the mean of 5 randomly selected cells in the field of view. *p < 0.05; unpaired *t*‐test with Welch's correction.

As a first step in determining the organization of intercellular signaling and interactions between cells, the morphology and connectivity of endothelial cells in *en face* veins and arteries were examined. AECs are highly elongated along the axis of blood flow (Figure 2A). In contrast, VECs are polygonal, non‐directional structures (Figure 2B). VECs had a significantly larger cell area and smaller length‐to‐width ratio than AECs, indicating that AECs were more elongated than VECs (Figure 2C). Interestingly, the number of neighbors of each AEC (6.0 ± 0.1, Figure 2C) is significantly higher than that for a VEC (5.1 ± 0.2). This reduction in the number of neighbors in the venous endothelium alters overall connectivity and may affect how VECs communicate across relatively large distances when compared to AECs.

### 3.2 Muscarinic‐Evoked Ca^2+^ Signal Transduction Occurs via Frequency Modulation in Venous Endothelial Cells

Ca^2+^ signaling induced by agonists varied among neighboring cells within the arterial endothelium [10, 11]. Increasing agonist concentration evoked a graded increase in the number of cells activated and in the amplitude of the Ca^2+^ response in each cell in the arterial endothelium [9, 10, 11]. To determine whether the VECs exhibit a similar heterogeneous response to agonists, we conducted concentration‐response experiments using ACh. These experiments aimed to assess the variation in Ca^2+^ signaling among venous endothelial cells and compare it to the observed patterns in the arterial endothelium.

**FIGURE 3:**
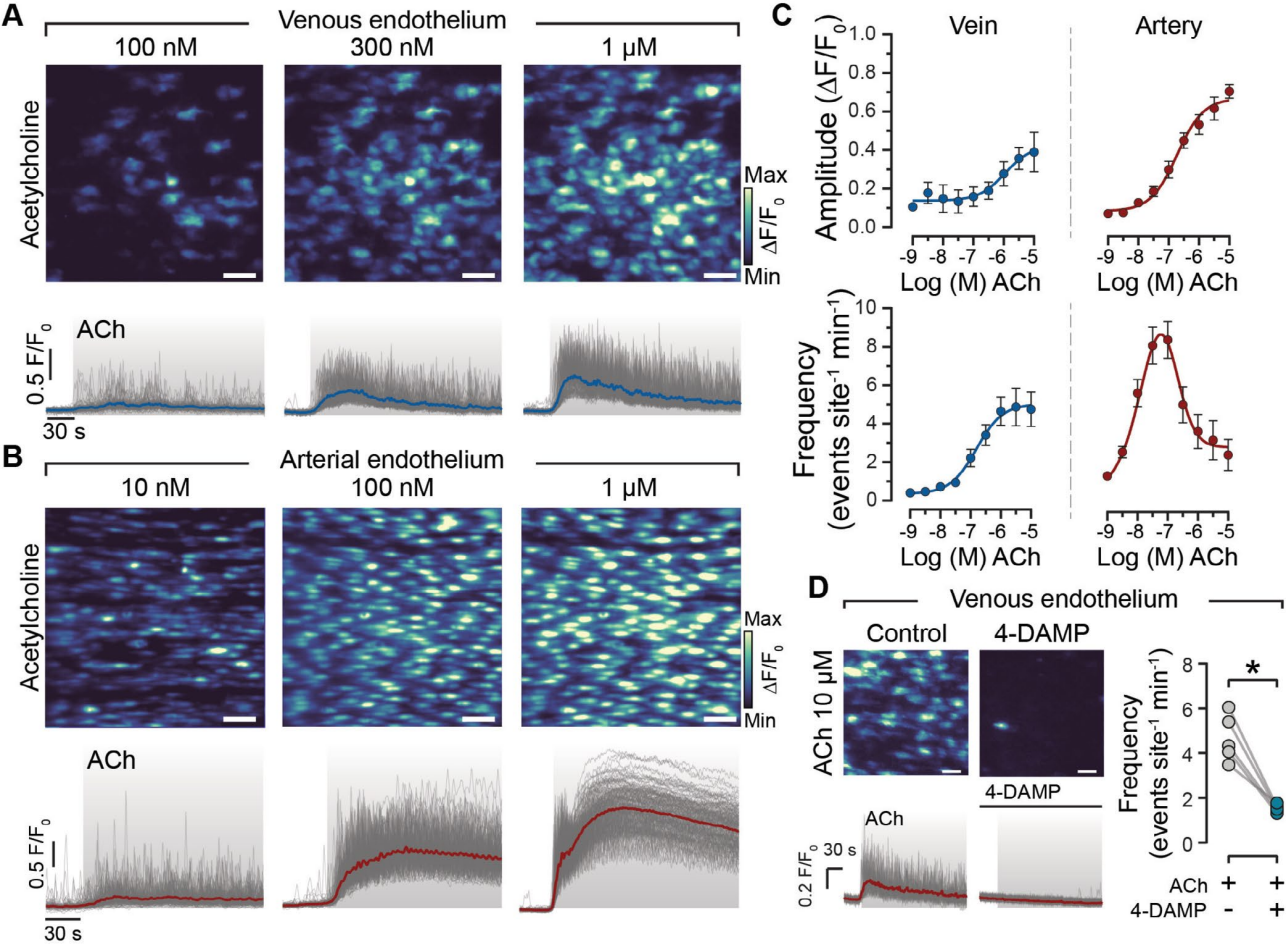
Acetylcholine concentration response in the venous and arterial endothelium. Ca^2+^ activity images from the same field of venous (A) and arterial (B) endothelium to increasing concentrations of acetylcholine (ACh). The corresponding Ca^2+^ traces from each endothelial cell in the field are shown below the panel. Each gray line indicates the Ca^2+^ signal from an individual endothelial cell, and the colored line represents the mean of all cells. The gray box indicates the period when ACh was present. (C) Concentration‐response curves showing the amplitude (top) and frequency (bottom) of Ca^2+^ events in response to increasing concentrations of ACh in veins (blue) and arteries (red). Data are shown as mean ± S.E.M. (*n* = 5). (D) Composite Ca^2+^ images and corresponding Ca^2+^ traces to ACh (10 μM) in the absence (left) and presence (middle) of the M_3_ receptor blocker 4‐DAMP (1 μM). Paired summary data of Ca^2+^ events (right), *n* = the number of animals. *p < 0.05; paired Student *t*‐test. Scale bars, 50 μm.

In the venous endothelium, a low concentration of ACh elicited a distributed, heterogeneous Ca^2+^ response across the field of endothelial cells. Some cells showed high activity while other cells were inactive (Figure 3A, Supplementary Figure S1A). Unlike AECs, where ACh prompted a concentration‐dependent increase in Ca^2+^ signal amplitude (Figure 3B,C), VECs showed only a modest increase in signal amplitude with rising ACh concentrations that occurred only after the frequency response had reached its maximum. The primary concentration‐dependent effect in VECs was an increase in the frequency of Ca^2+^ events (Figure 3A,C).

In AECs, although Ca^2+^ event frequency also increased, this effect saturated at concentrations above 100 nM, making further frequency changes undetectable (Figure S1B). This frequency saturation in AECs occurred at concentrations lower than the EC_50_ for the amplitude of the Ca^2+^ response (282.4 nM; 95% confidence interval, 134.3–609.6 nM). These findings suggest that AECs rely primarily on Ca^2+^ signal amplitude to achieve a graded response above the EC_50_ concentration, whereas VECs primarily use frequency modulation of Ca^2+^ events to encode response intensity.

The muscarinic M_3_ receptor blocker 4‐DAMP (100 nM) abolished the Ca^2+^ response to ACh (10 μM) in VECs (Figure 3D), confirming that ACh‐evoked Ca^2+^ signaling in VECs is mediated by M_3_ receptor activation. Our previous studies found that ACh‐induced Ca^2+^ responses in AECs also occur through M_3_ receptor activation [11].

Collectively, these findings demonstrate that although M3 receptor activation mediates ACh‐induced Ca^2+^ responses in both VECs and AECs, the venous and arterial endothelium utilize distinct signaling mechanisms to process information within the endothelium. Specifically, these results suggest that the venous endothelium may preferentially use frequency modulation, rather than amplitude, of Ca^2+^ signals to translate extracellular information.

### 3.3 Bradykinin‐Evoked Ca^2+^ Signal Transduction Occurs via Frequency Modulation in Venous Endothelial Cells

**FIGURE 4:**
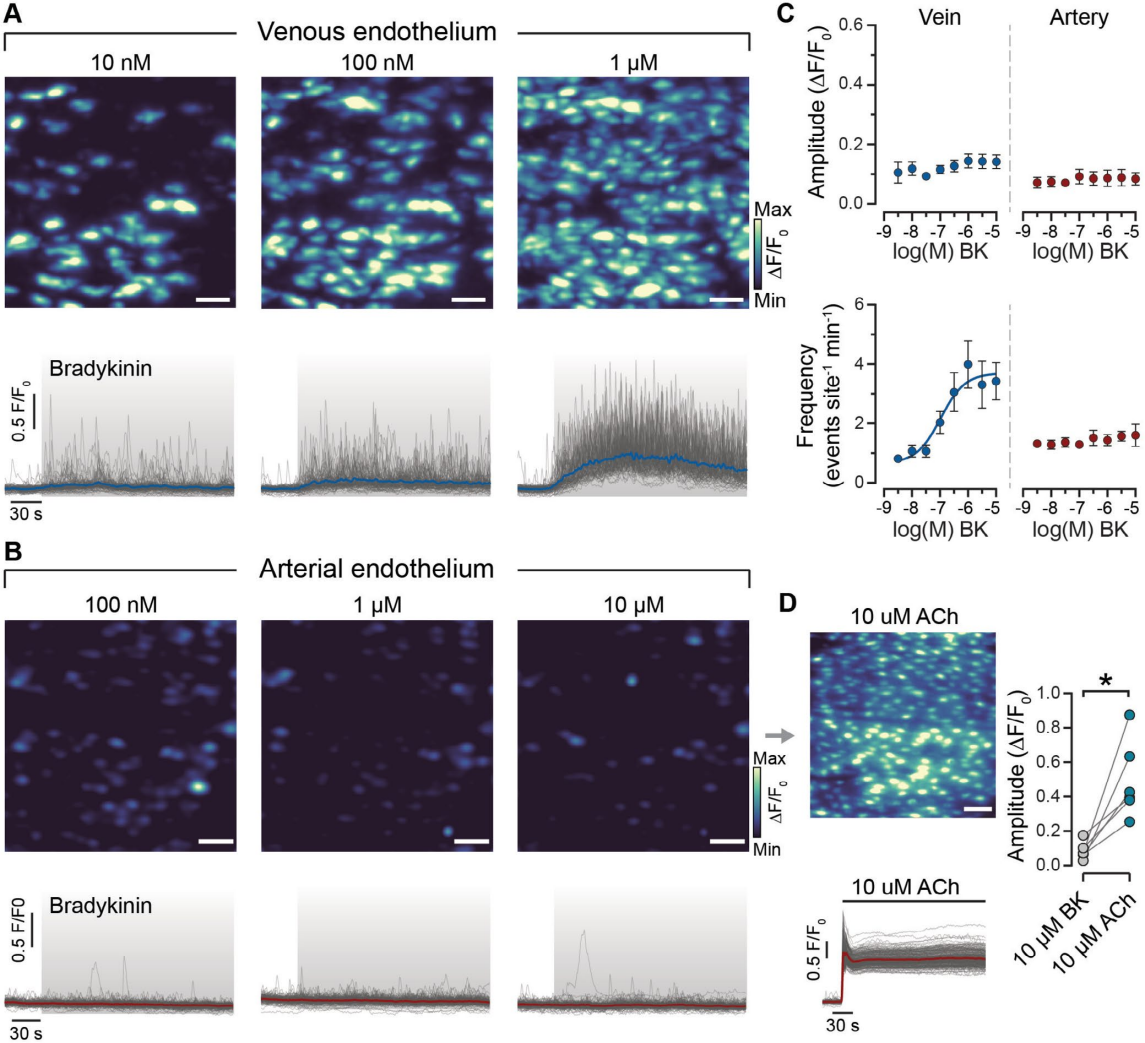
Bradykinin concentration‐response relationships in the venous and arterial endothelium. Ca^2+^ activity images from the same field of venous (A) and arterial (B) endothelium to increasing concentrations of bradykinin (BK). The corresponding Ca^2+^ traces from each endothelial cell are shown below the panel. Each gray line indicates the Ca^2+^ signal from an individual endothelial cell, and the colored line represents the mean of all cells. The gray box indicates the period when bradykinin was present. (C) Concentration‐response curves of the amplitude (top) and frequency (bottom) of Ca^2+^ events with increasing concentration of bradykinin in veins (blue) and arteries (red). Data are shown as mean ± S.E.M. (*n* = 5). (D) Composite Ca^2+^ image showing Ca^2+^ activity in the same field of endothelium shown in *B* to 10 μM acetylcholine (ACh) and paired summary data of Ca^2+^ activity in response to bradykinin (10 μM) and ACh (10 μM), *n* = the number of animals. *p < 0.05; paired Student *t*‐test. Scale bars, 50 μm.

To assess whether the observed differences in Ca^2+^ signaling mechanisms were unique to ACh or extended to other agonists, we next tested the response to the pro‐inflammatory mediator, bradykinin. Like ACh, bradykinin produced a heterogeneous Ca^2+^ response across the venous endothelium, with some cells showing high activity and others remaining quiescent (Figure 4A). In VECs, increasing bradykinin concentrations resulted in a concentration‐dependent rise in the frequency of Ca^2+^ events (Figure 4A,C). Notably, the amplitude of the Ca^2+^ events did not follow increasing bradykinin concentrations. These results indicate that VECs predominantly utilize frequency modulation to encode information across various agonists.

Unexpectedly, bradykinin failed to elicit any detectable rise above baseline in either the amplitude or frequency of Ca^2+^ events in AECs, even at concentrations up to 10 μM (Figure 4B). ACh (10 μM) evoked a Ca^2+^ response in the endothelium of the same arteries that were unresponsive to bradykinin (Figure 4D).

### 3.4 Network Analysis Reveals the Spatial Organization of Venous Endothelial Ca^2+^ Responses

The responses to both ACh and bradykinin revealed a highly non‐uniform pattern of cell activity in which there appeared to be localized clusters of cells that were sensitive to each agonist. We employed network analysis to quantify the spatial distribution and extent of interactions of Ca^2+^ signaling among venous endothelial cells. The initial goal was to determine whether the spatial arrangement of cells activated by ACh and bradykinin differed from the pattern expected if the activated cells were distributed randomly. By mapping the location and connectivity of the response of individual cells to stimulation, we sought to identify any inherent structure or clustering in cellular activation, which may suggest coordinated signaling and intercellular communication within the endothelial network.

**FIGURE 5:**
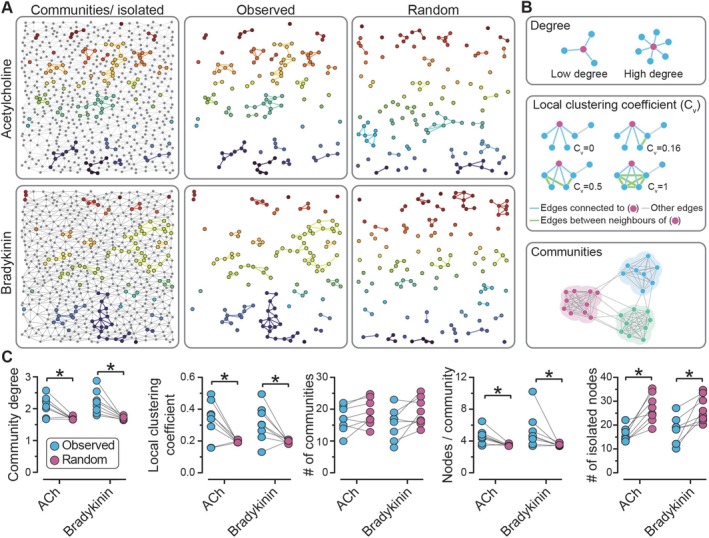
Network analysis of the spatial distribution of agonist‐specific sensing cells in the venous endothelium. (A) Network topology of the same field of venous endothelial cells in response to activation with acetylcholine (ACh, EC_20_, top) and bradykinin (EC_20_, bottom). Each dot represents an individual endothelial cell, with lines indicating connections to neighboring cells. Active cells are marked with colored dots, and colored lines connect neighboring cells that also respond. Each color represents a different community or isolated node. Random networks were generated for comparison with the observed connectivity. (B) Schematic illustrating various measurable network parameters. (C) Summary data presenting measurable network parameters from ACh‐ and bradykinin‐responsive cells across the venous endothelium, compared to those from a random distribution. Data represent *n* = 8 independent experiments from different animals; *p < 0.05, paired Student *t*‐test.

Network analysis of the most responsive VECs revealed significant differences in cellular organization and communication within the same endothelial field. Distinct subpopulations emerged when comparing the top 20% of cells ranked by response frequency to ACh versus bradykinin. These unisensitive subpopulations formed spatially distinct communities within the endothelial network (Figure 5A). The degree of local clustering, a metric that quantifies the number of connections each cell establishes within a community, was significantly higher in the measured network than occurred in random networks for both ACh and bradykinin (Figure 5B,C). Similarly, the local clustering coefficient, which reflects the interconnectivity of neighboring cells within a cluster, was also markedly greater in the measured networks (Figure 5B,C). These results indicate that the most responsive endothelial cells to either agonist aggregate into tightly connected communities, rather than being randomly distributed across the endothelium.

The observed networks for both ACh and bradykinin contained a similar number of distinct communities compared to their random counterparts. However, the size of each community—measured by the number of cells (nodes) per community—was significantly larger in the observed network (Figure 5A–C). Additionally, fewer isolated nodes (cells that responded to an agonist but did not belong to any community) were present in the observed network compared to random networks for both ACh and bradykinin (Figure 5A–C).

Collectively, these results indicate that the most responsive venous endothelial cells to ACh and bradykinin aggregate into larger, cohesive communities, rather than functioning independently in response to agonist stimulation. This clustering suggests a high degree of intercellular communication within the endothelial network, which likely facilitates communication and synchronized signaling to enhance the coordinated functional responses of endothelial cells to stimuli.

### 3.5 Venous Endothelial Cell Populations Show Limited Overlap in Response to ACh and Bradykinin

**FIGURE 6:**
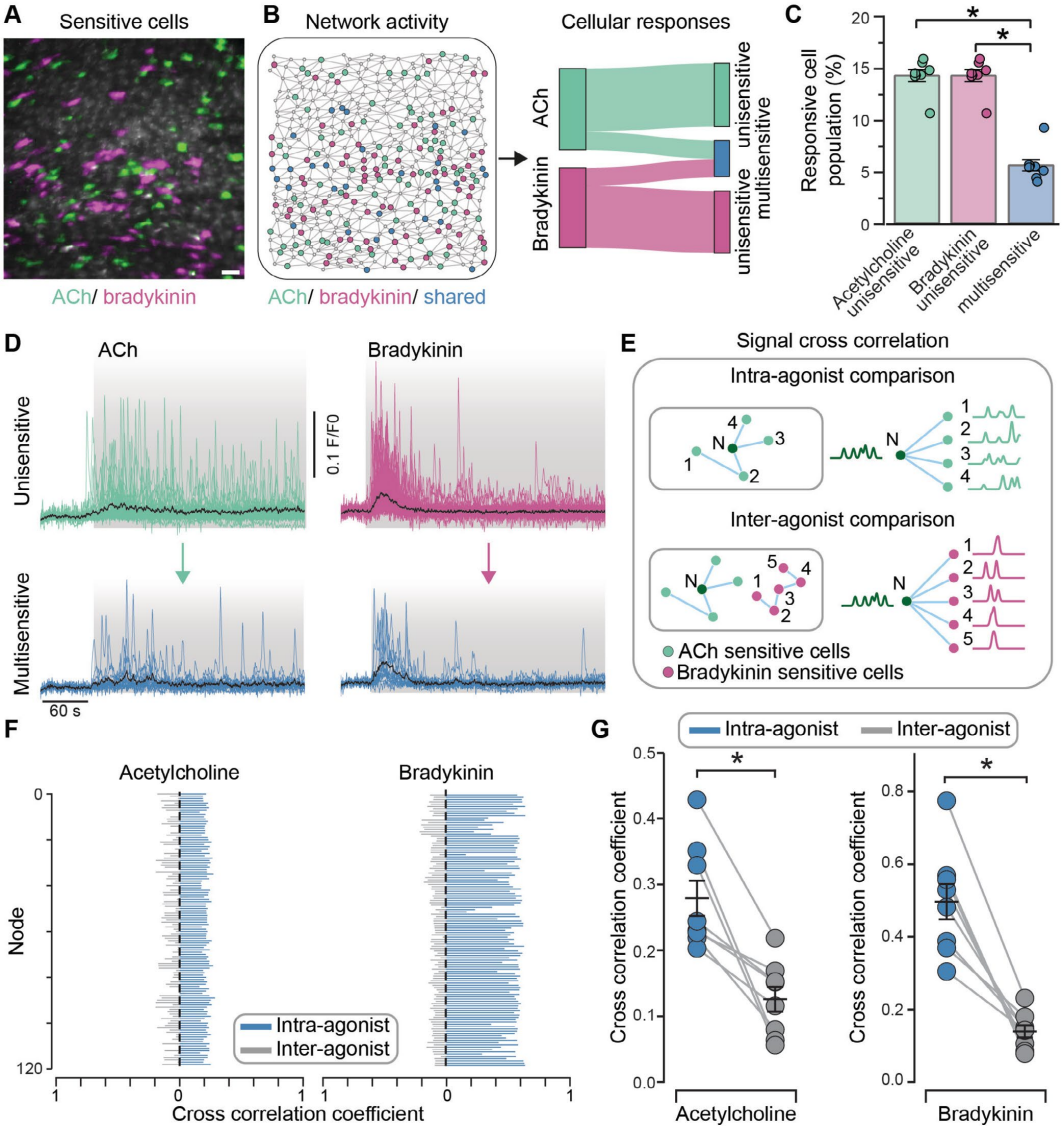
Spatial distribution of venous cellular responses to acetylcholine and bradykinin. (A) Composite Ca^2+^ image of mesenteric venous endothelium showing responses to EC_25_ concentrations of acetylcholine (ACh, green) and bradykinin (magenta). Cells responding to both agonists are depicted in white. (B) Network topology (left) and Sankey plot (right) showing cells responding exclusively to one agonist (unisensitive) or both (multisensitive). (C) Summary data showing the percentage of the total cell population that responded exclusively to either agonist or both. (D) Ca^2+^ signals from the most responsive cells to ACh or bradykinin, grouped by those that respond exclusively to the agonist (unisensitive) or both agonists (multisensitive cells). Black line represents the average response. (E) Schematic of cross‐correlation analysis comparing signals from a cell N with other ACh‐responsive cells (intra‐agonist, green) or bradykinin‐responsive cells (inter‐agonist, magenta). (F) Cross‐correlation coefficients for intra‐agonist (blue) versus inter‐agonist (gray) comparisons. (G) Summary of cross‐correlation coefficients for intra‐ versus inter‐agonist Ca^2+^ signals. Data represent *n* = 8 independent experiments from different animals; *p < 0.05, paired Student's *t*‐test.

The results thus far indicate that distinct subpopulations of endothelial cells respond to ACh and bradykinin, forming separate communities. We next quantified the extent of overlap of endothelial cells that were responsive to both ACh and bradykinin. Our analysis revealed that cells responsive to ACh largely exhibited exclusivity; there was minimal overlap observed in cells that also responded to bradykinin (Figure 6A–C). Similarly, bradykinin‐sensitive cells also exhibited a similar pattern of exclusivity (Figure 6A–C). However, while the populations of cells that were sensitive to each agonist were largely exclusive (hereafter referred to as unisensitive cells), there was a small number of cells (~5% of the global population) that responded to both ACh and bradykinin (multisensitive cells). These results demonstrate that distinct endothelial subpopulations are selectively activated by different agonists, highlighting the specialized roles and specific responses within the endothelial cell population.

In response to ACh and bradykinin, distinctive Ca^2+^ signals were generated in the cells activated by each agonist. The response to ACh was characterized by repeating Ca^2+^ oscillations that were approximately uniform throughout the activation period (Figure 6D). In contrast, while the response to bradykinin also exhibited repeated Ca^2+^ oscillations, activity was much more pronounced during the initial phase of activation and diminished with time (Figure 6D). This raises the question of whether the difference in response to ACh and bradykinin is due to the properties of the agonists themselves or to variations in the particular endothelial cells activated.

To address this question, we focused on the small population of cells (~5% of the entire population) that responded to both ACh and bradykinin. Significantly, there were no notable differences in the signaling characteristics of cells that exclusively responded to one agonist when compared to those that responded to both (Figure 6D). Cross‐correlation analysis showed that signals from cells activated by a specific agonist were more strongly correlated with signals from other cells within the same agonist‐responsive group (intra‐agonist correlation) than with signals from cells in the other agonist‐responsive group (inter‐agonist correlation, Figure 6E–G).

Collectively, these findings suggest that distinct subsets of venous endothelial cells respond to each activator and that these cells exhibit specialized responses to ACh and bradykinin. The limited overlap in cell activation and the unique signal correlation patterns within each agonist‐responsive population highlight the functional specificity of venous endothelial cell subpopulations in response to different stimuli.

### 3.6 Spectral Graph Theory and Betweenness Centrality Highlights the Central Role of a Small Population of Cells in Signal Integration

**FIGURE 7:**
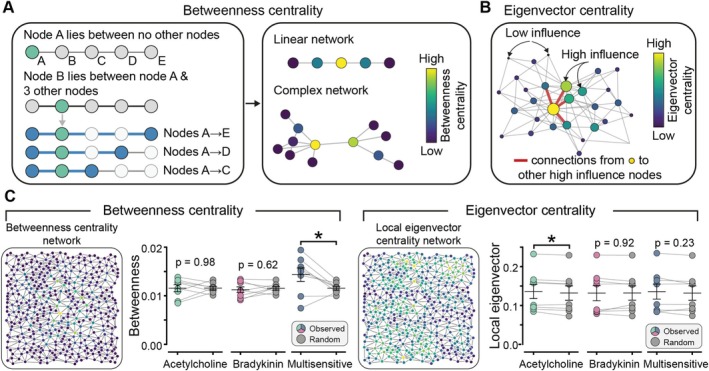
Strategic positioning of agonist‐responsive cells in venous endothelial networks. (A) Schematic illustrating betweenness centrality (measures how often a node serves as an intermediary between other node pairs) (B) Schematic illustrating eigenvector centrality (quantifies influence based on connections to highly connected nodes). (C) Network topology maps show betweenness centrality (left) and eigenvector centrality (right) in venous endothelium, where each node (dot) represents an individual endothelial cell, and lines represent edges (connections) between neighbors. Dot plots quantify centrality measures for cells responding exclusively to acetylcholine (green dots), exclusively to bradykinin (pink dots), or to both stimuli (blue dots, multisensitive), compared to random distributions (gray dots). Data represent *n* = 8 independent experiments from different animals; *p < 0.05, paired Student's *t*‐test.

We next investigated the roles of cells within the various agonist‐sensitive subpopulations in signal propagation and integration. To do this, we focused on assessing the importance of their positions within the network using key metrics for studying information flow and propagation: betweenness centrality and eigenvector‐based centralities (Figure 7A,B).

In network analysis, betweenness centrality quantifies how often a node (cell) lies on the shortest paths between pairs of other nodes, identifying nodes that serve as critical intermediaries in the network. Nodes with high betweenness centrality are often positioned at key junctions, acting as bridges for information flow. Our results show that only cells responding to both agonists (multisensitive cells) exhibited significantly higher betweenness centrality than expected from random chance (Figure 7A,C). In contrast, unisensitive cells showed no significant difference from random distributions. This finding suggests that multisensitive cells are uniquely positioned as communication bridges, facilitating signal transfer between otherwise distinct ACh‐responsive and bradykinin‐responsive communities within the endothelial network. By occupying these strategic interface positions, multisensitive cells enable cross‐talk and coordinated signaling between specialized subpopulations without themselves serving as dominant local influencers.

Eigenvector‐based centralities quantify a node's ability to receive and transmit information across the network at local and global scales. We first examined the influence of cells within their local neighborhood. Our results show that only cells responding selectively to ACh exhibited significantly higher local eigenvector centrality compared to randomly distributed cells (Figure 7B,C). Bradykinin‐selective and multisensitive cells showed no significant difference from random distributions. This finding suggests that ACh responsiveness forms concentrated local hotspots where ACh‐sensitive cells cluster around locally influential neighborhoods—regions where neighboring cells are themselves highly connected and influential within their immediate vicinity.

We next examined the spread of information at a global scale (Figure S2). Our results reveal that none of the agonist‐responsive cell populations—ACh‐selective, bradykinin‐selective, or multisensitive cells—exhibited significantly different global eigenvector centrality compared to random distributions. This suggests that endothelial responses to these agonists are predominantly modular rather than system‐wide, with activation patterns localized to specific network domains rather than recruiting cells positioned at the global core of the vascular network. However, because the analysis was restricted to the top 20% of responding cells, sensitivity to global network properties may have been reduced, potentially leading to an underestimation of system‐wide influences.

Collectively, these results illustrate distinct organizational principles for different cell subpopulations in the endothelial network. Multisensitive cells function as specialized communication bridges that integrate signals between ACh and bradykinin signaling domains through their strategic positioning along critical network pathways. ACh‐responsive cells organize into locally influential clusters that enable efficient signal amplification within defined tissue regions. This modular architecture supports both specialized local responses and coordinated inter‐domain communication, revealing how the endothelial network achieves complex signal processing through complementary organizational strategies. This modular, hierarchically organized architecture with functionally specialized subpopulations and strategic connectivity, illustrates that the endothelium is more analogous to neural circuit architectures in the brain, than a uniform sheet of identical cells [35].

### 3.7 Neighboring Cell Activity in the Venous Endothelium Modulates Ca^2+^ Event Probability but Not Event Characteristics

**FIGURE 8:**
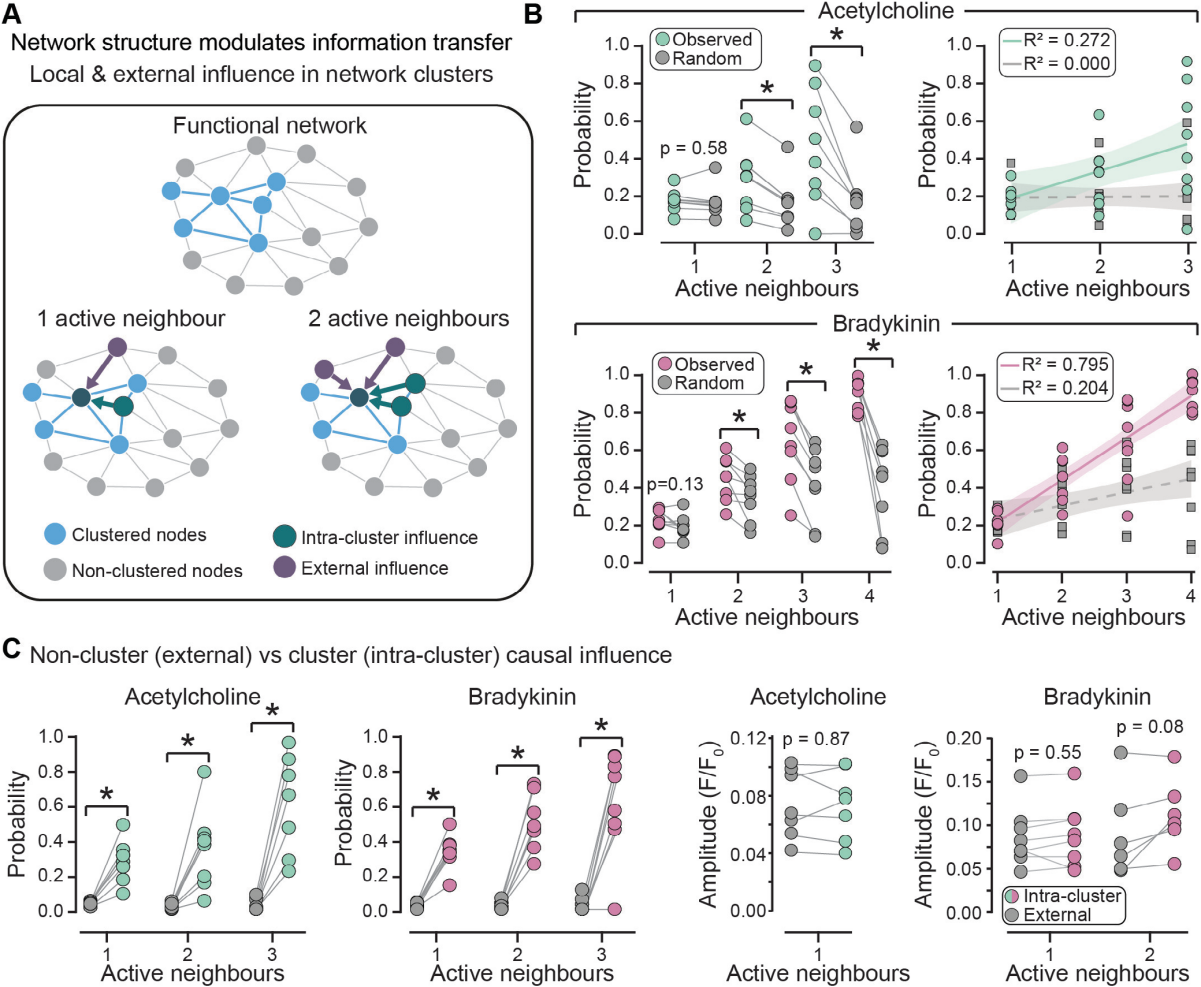
Causal influence of neighboring cell activity on agonist‐evoked Ca^2+^ events in the venous endothelium. (A) Schematic illustrating a functional network with clustered nodes (blue) and the influence of neighboring cells that are part of the same functional cluster (intra‐cluster influence, green) or not part of the cluster (external influence, purple). Active neighbors are defined as cells responding within three times the average full duration at half maximum (FDHM) of all Ca^2+^ events. (B) Probability plots (left) show the likelihood of Ca^2+^ events occurring with increasing number of active neighbors for all events (10 min recording). Random data (gray dots) generated by shuffling event times while maintaining a cells total number of events and neighbor relationships. Linear regression curves (right) increasing number of active neighbors for the measured and random data. (C) Intra‐cluster versus external influence. Probability (left) and amplitude (right) plots of the Ca^2+^ response in target cell with increasing number of active neighbors for external influence (gray dots) versus intra‐cluster (colored dots). Data represent *n* = 8 independent experiments from different animals; *p < 0.05, paired Student's *t*‐test.

Having shown that specialized clusters and hubs give the endothelial network a neuronal‐like organization, we next asked how activity within local communities shapes the behavior of individual cells (Figure 8A). To assess this, we quantified the probability of a Ca^2+^ event occurring in a cell, as well as the characteristics of these events, based on the number of neighboring cells that responded within a defined time window. This time window was set to three times the median full duration at half maximum (FDHM) of all Ca^2+^ events, to capture the complete Ca^2+^ response while accounting for variability in individual event duration.

To test for causal influence, we performed a permutation analysis in which events were randomly reassigned across all active cells, while preserving the observed number of events per cell and network structure. This randomization was repeated 100 times. Compared to the permutation dataset, the measured data showed a significantly greater probability of an event occurring, for both ACh and bradykinin, with a clear linear relationship between the number of active neighbors and event probability (Figure 8B). In contrast, no such influence was observed for the characteristics of the Ca^2+^ events (amplitude, rise time, fall time, or FDHM), indicating that neighboring activity affects event likelihood but not the intrinsic properties of individual responses (Figure S2). Thus, neighboring activity acts as a trigger that increases the likelihood of Ca^2+^ events, without altering the fundamental properties of the responses themselves.

We next tested whether this influence depended on the functional relationship between a cell and its neighbors—specifically, whether neighbors belonging to the same cluster (intra‐cluster influence) had a stronger influence than those outside the functional cluster (external influence) (Figure 8A). This analysis revealed that the probability of a Ca^2+^ event occurring was significantly higher, for both ACh and bradykinin, when active neighbors were part of the same functional cluster as the target cell (Figure 8C). This effect was consistent across conditions where one, two, or three neighbors were active. Thus, functional clustering establishes preferential communication pathways within the venous endothelial network, such that cells sharing similar agonist sensitivities exert stronger intercellular influence than functionally distinct neighbors. The functional relationship of the neighbors had no significant effect on the characteristics of the Ca^2+^ response (amplitude, rise time, fall time, and FDHM) (Figure 8D, Figure S2).

Together, these findings demonstrate that neighboring cell activity modulates the probability of Ca^2+^ events in venous endothelium, with intra‐cluster neighbors exerting the strongest influence, while the kinetic properties of Ca^2+^ signals remain cell‐intrinsic and unaffected by local network activity.

### 3.8 Relationship Between Ca^2+^ and Vasodilation in the Arterial and Venous Endothelium

Having established how neighboring activity shapes Ca^2+^ signaling within endothelial networks, we next examined how these cellular dynamics translate into functional vascular responses. To investigate the relationship between endothelial Ca^2+^ signaling and vasodilation, the vasoreactivity of arteries and veins pre‐contracted with low concentrations of phenylephrine (100 nM–1 μM) was assessed. The phenylephrine concentration was titrated to achieve a ~20% decrease in vessel diameter [27]. Once a stable contraction was maintained for 30 min, the responses to cumulative concentrations of ACh or bradykinin were measured. ACh induced a concentration‐dependent relaxation in arteries and veins (Figure S3). Similarly, bradykinin also caused the relaxation of pre‐contracted veins.

**FIGURE 9:**
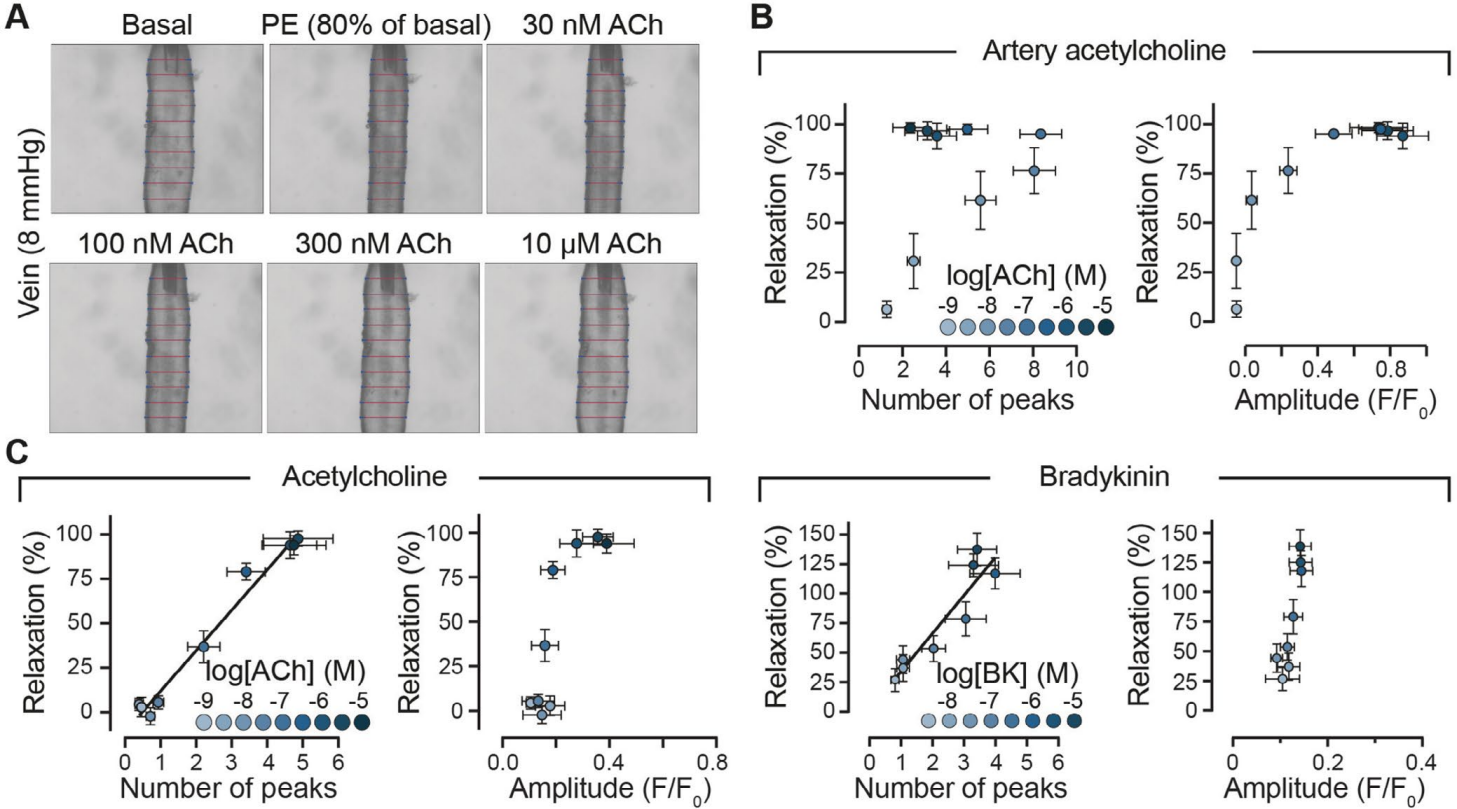
Vasoreactivity of arteries and veins to acetylcholine and bradykinin. (A) Images showing an isolated, cannulated and pressurized vein. Pressurized arteries (70 mmHg) and veins (8 mmHg) were contracted, using phenylephrine, to 80% of their resting diameter, before the addition of acetylcholine (ACh) or bradykinin. (B) Relationship between the amplitude of Ca^2+^ events and arterial relaxation to ACh. (C) Relationship between the frequency of Ca^2+^ events or amplitude of Ca^2+^ events and venous relaxation in response to ACh (left) and bradykinin (right). Data re‐analyzed from experiments shown in Figures 3 and 4, and Figure S1.

To further explore the relationship between endothelial Ca^2+^ signaling and endothelium‐dependent relaxation, we re‐analyzed the experiments presented in Figure 3, Figure 4, and Figure S3. Our analysis revealed a linear relationship between the frequency of Ca^2+^ events and venorelaxation, indicating that a higher frequency of Ca^2+^ events is associated with greater venous relaxation (Figure 9). In contrast, the amplitude of Ca^2+^ events did not exhibit the same positive linear relationship in the venous endothelium.

These findings suggest that the frequency of Ca^2+^ events plays a more crucial role in regulating venous relaxation than the amplitude of the signals, further underscoring the distinct signaling mechanisms at play in the venous endothelium.

## 4 Discussion

In this study, we demonstrate that endothelial cells are organized into distinct subsets, each forming specialized communities that respond selectively to ACh and bradykinin. Within these communities, activation of neighboring cells increases the likelihood of a cell responding, leading to localized areas of heightened responsiveness. Notably, there is minimal overlap in the sensitivity of these communities to other activators, indicating that each subset is tuned to specific stimuli. We also demonstrate that distinct activators elicit unique signaling patterns, which are determined by the specific agonist rather than being inherent properties of the endothelial cell subpopulations. These findings reveal the functional specificity of endothelial cell subpopulations in response to diverse stimuli.

Despite the overall specialization, about 5% of the global cell population responded to ACh *and* bradykinin. This small group of multisensitive cells occupied critical bridging positions within the endothelial network, exhibiting significantly higher betweenness centrality than randomly distributed cells. These cells function as communication bridges that facilitate signal transfer between otherwise distinct ACh‐responsive and bradykinin‐responsive communities. By occupying strategic positions along the shortest pathways connecting different functional domains, multisensitive cells enable cross‐talk and coordinated signaling between specialized subpopulations, helping to integrate localized responses across the endothelial network. Thus, a small population of multisensitive bridge cells holds disproportionate influence over endothelial communication, acting as strategic hubs that integrate otherwise segregated signaling domains and thereby sustain coordinated vascular function.

In transducing information, we also demonstrate that venous endothelial cells predominantly encode extracellular activation through changes in intracellular Ca^2+^ signal frequency, whereas arterial endothelial cells rely primarily on the amplitude of Ca^2+^ signals. This difference in encoding mechanisms highlights the distinct Ca^2+^ signaling strategies employed by different vascular beds, revealing functional specialization within the cardiovascular system.

### 4.1 Network Analysis and Cell Communication

In the present study, we demonstrate the complexity and heterogeneity of endothelial Ca^2+^ signaling across the vascular system. Using network analysis to map interactions between individual cells, we show that venous endothelial cells organize into distinct stimulus‐specific communities. These communities were characterized by significantly higher clustering coefficients than expected from random networks, indicating that agonist‐responsive cells form tightly interconnected neighborhoods rather than being diffusely distributed. Such organization enhances local communication and coordination, allowing groups of cells to respond collectively and efficiently to stimulation.

Clustering confers several functional advantages: it enables collective sensing, allowing cells to detect and process multiple independent inputs, promotes robust signal propagation, and provides resilience by allowing neighboring cells to compensate for inactive or damaged partners [36]. Understanding these clustering dynamics is essential, as they reveal how local endothelial networks manage responses to stimuli. Additionally, localized clustering also supports region‐specific vascular control without requiring global activation [37]. Disruptions in such networks may underlie endothelial dysfunction and vascular diseases, as occur in other biological systems [38], making network analysis a valuable tool for understanding pathology and informing therapeutic strategies. This functional specialization, which was also observed in the arterial endothelium [9, 10, 11], allows the endothelium to fine‐tune responses to varying physiological cues.

### 4.2 Spectral Graph Theory and Betweenness Centrality: Analysis in Signal Integration and Communication in the Venous Endothelium

The formation of distinct communities enables the management of diverse external stimuli. However, the organization relies on effective intercellular communication to synchronize activity both within and between these communities. This communication is crucial for vascular function, where coordinated responses are necessary for efficient vasodilation and blood pressure regulation. To identify cells critical for such coordination, we examined betweenness and eigenvector centrality.

Our spectral graph theory analysis revealed that venous endothelial signaling is modular, with distinct subpopulations contributing differently to network organization. Multisensitive cells exhibited elevated betweenness centrality, positioning them as bridges between ACh‐ and bradykinin‐responsive domains. By linking otherwise separate subnetworks, these cells are well placed to integrate signals and coordinate activity across communities. In contrast, ACh‐selective cells showed higher local eigenvector centrality, forming influential neighborhoods that amplify local signaling. Bradykinin‐selective cells did not display enrichment in either local or global centrality, consistent with a more distributed activation pattern. None of the responsive groups exhibited elevated global eigenvector centrality, suggesting that agonist responses in the venous endothelium are organized in modular, rather than system‐wide, patterns.

The divergence between ACh and bradykinin likely reflects their distinct physiological roles. ACh acts as a rapid vasodilator, where clustered hotspots of responsiveness permit focal and spatially restricted control of vascular tone. Bradykinin, by contrast, is linked to barrier regulation, inflammation, and fluid balance—processes that benefit from diffuse activation across the endothelial layer. This arrangement suggests a division of labour: ACh signaling is optimized for local amplification, bradykinin for distributed signaling control, and multisensitive cells for bridging information between domains. Together, these features define a modular communication strategy that balances local specialization with cross‐community integration, providing resilience and flexibility in vascular control.

### 4.3 Influence of Neighboring Cell Activity

The synchronization of Ca^2+^ responses within stimulus‐specific communities depends on robust local communication. To further dissect this organization, we examined how neighboring cell activity influences endothelial Ca^2+^ dynamics, offering mechanistic insight into how functional communities shape signaling at the single‐cell level. Our findings reveal that endothelial Ca^2+^ dynamics in venous networks are strongly shaped by the activity of neighboring cells, but in a selective manner. The probability of a Ca^2+^ event increased in proportion to the number of active neighbors, whereas the characteristics of individual events—amplitude, rise time, fall time, and FDHM—remained unaffected. This indicates that intercellular communication within a defined community in the endothelium regulates the likelihood of cellular activation but not the intrinsic properties of the Ca^2+^ response once initiated. Such a separation of control is consistent with a model in which intercellular signals, transmitted via inside‐out and outside‐in signaling or paracrine messengers [39, 40], alter the threshold for event initiation, while intracellular mechanisms govern the kinetics of the Ca^2+^ response. This selective influence on event probability rather than event characteristics provides additional evidence that the venous endothelium employs frequency‐based encoding of extracellular signals, where intercellular communication modulates the rate of Ca^2+^ events while preserving the amplitude and kinetics that characterize individual responses.

A further layer of organization was revealed by our cluster analysis. Cells sharing the same functional cluster exerted a stronger influence on their neighbors than cells outside the cluster, regardless of the number of active neighbors. This suggests that functional clustering creates preferential communication pathways within the endothelial network. Such pathways may allow for the emergence of sub‐regional domains of heightened sensitivity, ensuring that groups of functionally similar cells coordinate their activity more effectively than functionally distinct cells. Importantly, these findings align with the concept of endothelial heterogeneity, where subsets of endothelial cells exhibit distinct receptor profiles, signaling behaviors, and physiological roles.

Together, these findings reveal two key principles of endothelial communication: neighbor activity increases the probability of Ca^2+^ events, and this influence is stronger within functional clusters than cells outside them. This organization allows the venous endothelium to coordinate responses across the vessel wall while maintaining functional specialization within distinct cellular communities. By coupling event timing rather than event characteristics, the system preserves the intrinsic properties of individual Ca^2+^ responses while enabling network‐level coordination of cellular activity. This organization suggests that venous endothelial communication is not uniform but highly structured, balancing global coordination with local functional specialization.

This structured organization parallels principles of causal network inference in neuroscience, where effective connectivity—the causal influence one node exerts on another—depends on both anatomical proximity and functional relationships [35, 41, 42]. Like neural circuits that exhibit small‐world architectures with clustered local processing and strategic long‐range connections [30], the venous endothelium achieves efficient signal coordination through hierarchically organized communities linked by strategically positioned bridging cells.

### 4.4 Stimulus‐Specific Synchronization of Endothelial Ca^2+^ Signals

In addition to examining the spatial relationships among agonist‐sensing cells, we employed cross‐correlation analysis to measure the functional interactions in signaling dynamics among cells responsive to ACh and bradykinin. Cross‐correlation is a statistical technique used to evaluate the relationship between two signals over time [33]. The cross‐correlation analysis revealed that signals from cells activated by a specific agonist (e.g., ACh) were more correlated with signals from other cells within the same population than with signals from populations, within the same field of endothelium, responding to a different agonist (e.g., bradykinin). This finding suggests that there is a strong network of local communication and synchronization among cells responding to the same stimulus.

The results from our cross‐correlation analysis not only provide insights into the signaling dynamics of endothelial cells but also highlight the complexity of cellular communication within the vascular system. Understanding these interactions is vital for deciphering how endothelial cells contribute to overall vascular homeostasis and how these mechanisms can be targeted for therapeutic interventions in vascular disease.

### 4.5 Difference in Venous and Arterial Endothelial Cell Morphology

The structure of endothelial cells plays a crucial role in this communication process, as their morphology influences how they interact with one another and transmit signals. We observed that AECs are elongated and highly directional, aligning with the axis of blood flow while VECs exhibit a more polygonal and non‐directional morphology. The organization of VECs appears similar to that of AECs when the latter are observed in the absence of shear stress imposed by blood flow. Arterial walls experience shear stress ranging from 10 to 70 dynes/cm^2^, whereas in veins, the shear stress is between 1 and 6 dynes/cm^2^ [43, 44]. Under static conditions, AECs form a polygonal monolayer, but increased shear stress results in elongation and alignment with flow direction within 24 h [45]. It is tempting to speculate that the “cobblestone” or polygonal pattern observed in VECs arises from the low shear stress levels the cells experience [46].

The structural differences between arterial and venous endothelial cells, influenced by varying shear stress levels, may play a critical role in how these cells communicate, coordinate, and modulate vascular responses.

### 4.6 Agonist‐Evoked Ca^2+^ Signaling in the Venous Endothelium

Effective communication between a ligand and a cell requires a clear link between the external stimulus and the cell's internal response to ensure accurate information transmission. This occurs through information encoding, where a specific signal corresponds to a stimulus, triggering distinct physiological reactions. In endothelial cells, the timing and location of Ca^2+^ signals are crucial for conveying the necessary information to prompt a cellular response. Our results show that the response to agonist activation varies among cells, with more cells activating as the ligand concentration increases. Additionally, we found key differences in how venous and arterial endothelial cells respond to ACh and bradykinin within the activated cell populations.

### 4.7 Distinct Mechanisms of ACh‐Induced Vasodilation in Veins and Arteries

To explore these differences in more detail, we examined the mechanisms underlying ACh‐evoked responses. ACh is a potent vasodilator that plays a crucial role in regulating vascular tone and endothelial function [25, 47]. ACh‐evoked Ca^2+^ responses in venous endothelial cells are mediated by activation of the muscarinic M3 receptor, as shown by the M3 receptor blocker, 4‐DAMP. Our previous study demonstrated that ACh‐evoked Ca^2+^ responses in AECs are also mediated by M3 receptor activation [11]. Despite the involvement of M3 receptors in both venous and arterial endothelial cells, ACh‐induced Ca^2+^ responses differ significantly between these cell types, suggesting complex signaling mechanisms.

In veins, ACh‐induced dilation was associated with a concentration‐dependent increase in the frequency of Ca^2+^ responses, with a direct correlation between the frequency of Ca^2+^ events and the extent of dilation. This indicates that frequency is the primary signaling mechanism for dilation in veins. In contrast, arteries exhibit a concentration‐dependent increase in the amplitude of the Ca^2+^ response, but a bell‐shaped change in frequency as ACh concentration increased. This observation suggests that modulation of the amplitude of Ca^2+^ signaling plays a central role in vasodilation in arteries.

Interestingly, in AECs, the saturation of Ca^2+^ event frequency occurs at concentrations lower than the EC_50_ for the amplitude of the Ca^2+^ response. Arteries continue to dilate in a concentration‐dependent manner beyond the EC_50_ of the amplitude response, while the frequency of Ca^2+^ events decreases. This finding suggests that amplitude is the predominant signaling mechanism in arteries. We previously demonstrated this relationship between amplitude and vasodilation in mesenteric arteries from Wistar rats fed either a standard or high‐fat diet [38]. Together, these distinct patterns of Ca^2+^ signaling indicate that veins and arteries utilize different mechanisms to regulate vascular tone in response to ACh.

### 4.8 Differential Ca^2+^ Signaling in Venous and Arterial Endothelial Cells in Response to Bradykinin

Bradykinin is a potent vasodilator that increases blood flow during inflammation, aiding immune cell delivery and regulating vascular permeability [48, 49]. These actions facilitate immune cell recruitment, leading to the characteristic signs of inflammation, such as redness, heat, swelling, and pain.

Our study offers insights into the differential responses of mesenteric arterial and venous endothelial cells to bradykinin. In venous endothelial cells, there was a concentration‐dependent increase in the frequency of Ca^2+^ events, with minimal changes in amplitude. A direct correlation between event frequency and venodilation suggests that frequency modulation plays a key role in the venous response to bradykinin. In contrast, arterial endothelial cells exhibited no Ca^2+^ response to bradykinin, despite responding to ACh. The differential sensitivity of venous and arterial endothelial cells to bradykinin highlights the complexity of endothelial signaling and the specialized role of veins in inflammatory responses. Studies have shown that bradykinin significantly increases permeability in veins but not in arterioles [50]. This suggests that the modulation of Ca^2+^ response frequency is a crucial mechanism by which veins regulate vascular tone and permeability during inflammation, facilitating immune cell migration and tissue repair.

Our network analysis demonstrates how venous endothelial cells are organized into distinct communities with specialized hub cells that coordinate responses through strategic positioning within the network. The distinct morphological characteristics between VECs and AECs—with AECs displaying elongated, flow‐aligned architecture versus the polygonal morphology of VECs—indicate that network organization differs substantially between vessel types. These structural differences highlight how network architecture may influence signaling strategies across the vascular system. Our findings reveal how information propagates and coordinates within the venous endothelium; however, how arterial endothelial networks organize and whether this organization contributes to their distinct encoding strategy (amplitude vs. frequency modulation) requires further investigation. The encoding preferences we demonstrate likely stem from vessel‐specific differences in intracellular Ca^2+^ signaling machinery—including receptor coupling, IP_3_ dynamics, and Ca^2+^ handling proteins—with the potential interplay between network topology and molecular signaling mechanisms remaining unexplored. Although the precise reasons for the differences remain unclear, Ca^2+^ signal frequency and amplitude regulate distinct downstream signaling pathways, which may be central to the different functions of arteries and veins. Transient, repetitive Ca^2+^ spikes more effectively activate kinases like CaMKII and PKC, which act as integrators of spiking activity to fine‐tune gene expression and functional outcomes. In contrast, sustained, moderate Ca^2+^ elevations are better suited for processes such as prolonged contraction or activation of transcription factors like NFAT. Understanding how network architecture influences encoding mechanisms will require comparative analysis of both vessel types and investigation of the molecular determinants of Ca^2+^ signal encoding strategies, to reveal how structure and function integrate across the cardiovascular system.

## 5 Conclusion

This study highlights the complexity and variation of endothelial Ca^2+^ signaling between venous and arterial systems. While the heterogeneity of agonist‐evoked Ca^2+^ responses is well established in arterial endothelium [9, 10, 11], we show here for the first time that intact venous endothelium exhibits comparable heterogeneity. Venous endothelial cells assemble into distinct stimulus‐specific communities that enhance coordinated responses and are essential for vascular homeostasis. Within this organization, ACh‐sensitive cells function as local hubs, bradykinin‐responsive cells adopt a more diffuse communication strategy, and a small subset of multisensitive cells act as integrators bridging otherwise separate domains. Neighbor activity increased the probability of Ca^2+^ events without altering their kinetics, revealing a mechanism that promotes coordinated activation while preserving the integrity of single‐cell signaling. These results establish the endothelium as a modular signaling network, where specialized communities and rare integrator cells coordinate local and global responses essential for vascular homeostasis.

Comparisons between vascular beds further demonstrate distinct encoding strategies: veins primarily encode information through frequency modulation of Ca^2+^ events, whereas arteries rely more on amplitude modulation. This divergence reflects vascular bed–specific specialization, aligning with the roles of arteries in resistance control and veins in capacitance, permeability, and inflammatory regulation. Together, these findings provide a framework for understanding how endothelial network architecture and signaling diversity underpin vascular homeostasis, flexibility, and resilience, while offering new directions for investigating how disruption of these mechanisms contributes to vascular disease.

## Author Contributions

Conceptualisation, M.D.L., J.G.M., C.W.; Methodology, M.D.L., C.W., R.A.C., X.Z., P.U., C.B., J.G.M.; Investigation, M.D.L. and R.A.C.; Writing – original draft, M.D.L., J.G.M.; Writing – review and editing, M.D.L., J.G.M., C.W., X.Z., P.U., R.A.C., C.B.; Funding Acquisition, J.G.M., C.W., M.D.L., X.Z., C.B.

## Conflicts of Interest

The authors declare no conflicts of interest.

## Supporting information


**Figure S1:** Calcium responses to acetylcholine in the venous and arterial endothelium.
**Figure S2:** Global eigenvector—neighbor influence characteristics.
**Figure S3:** Vasoreactivity of mesenteric arteries and veins to acetylcholine and bradykinin.

## Data Availability

All study data are included in the article and Supporting Information.
