# Supplementary material for: Endothelial Cell Organization Drives Distinct Agonist‐Specific Ca2+ Dynamics in Arteries and Veins
Source: Acta Physiol (Oxf). 2025 Nov 17;241(12):e70132. doi: 10.1111/apha.70132 (PMC12621180; doi:10.1111/apha.70132)
Supplement: Supplementary file 1 — Figure S1: Calcium responses to acetylcholine in the venous and arterial endothelium. Figure S2: Global eigenvector—neighbor influence characteristics. Figure S3: Vasoreactivity of mesenteric arteries and veins to acetylcholine and bradykinin. [file APHA-241-e70132-s001.docx]

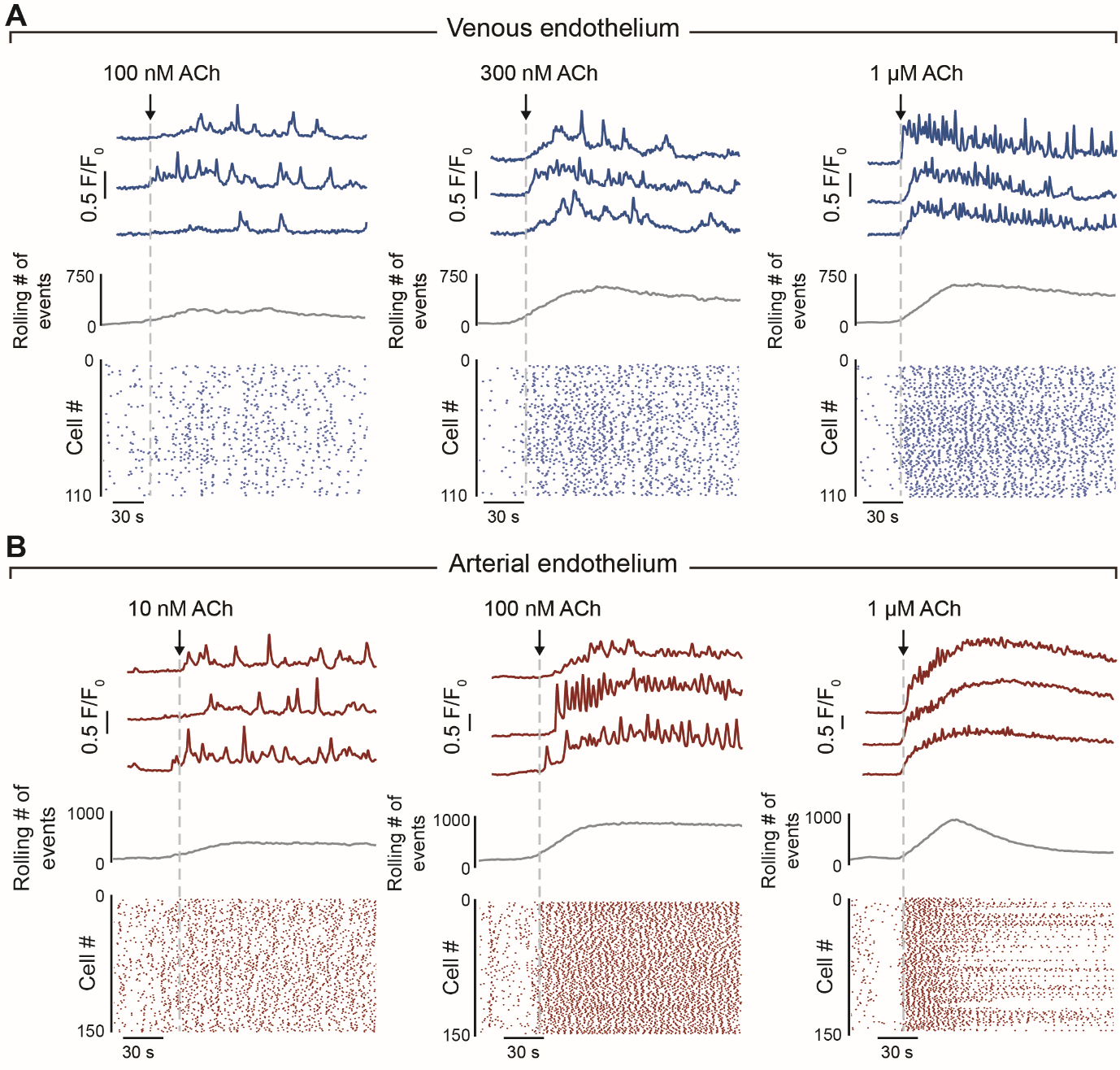


Supplementary Figure 1 – Calcium responses to acetylcholine in the venous and arterial endothelium.

(Top) Representative Ca²⁺ traces from three individual cells demonstrating dose-dependent responses to increasing acetylcholine (ACh) concentrations in venous (A) and arterial (B) endothelial cells. (Bottom) Raster plots show the temporal occurrence of peak Ca²⁺ events for all cells within the field of view, with each horizontal line representing an individual cell. The rolling average curve (middle) quantifies the population-level Ca²⁺ activity by displaying the mean number of events at each time point. Black arrows indicate ACh application timing. The dashed grey line provides temporal reference across Ca²⁺ traces, raster plots, and rolling event analysis.


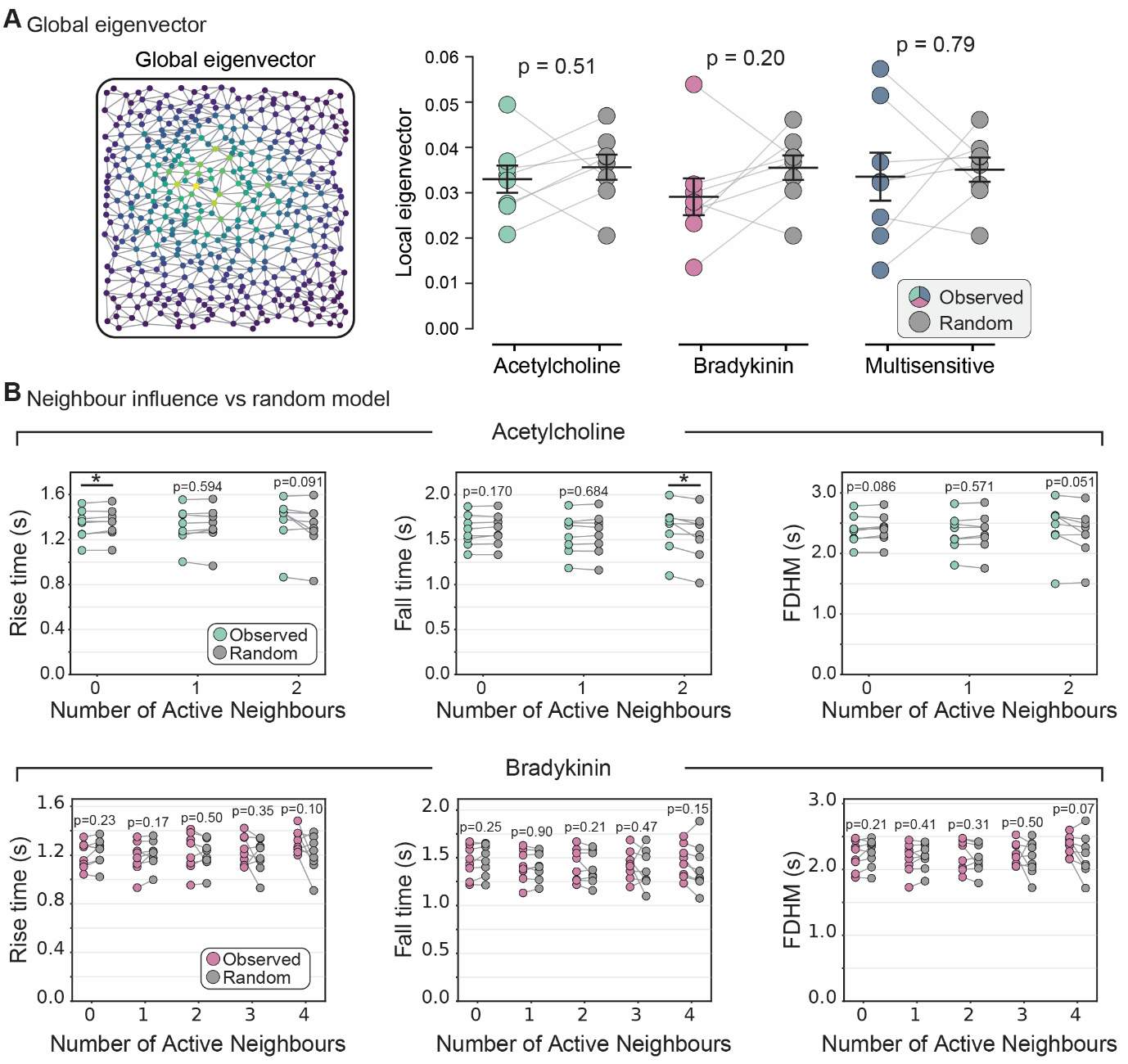


Supplementary Figure 2 – Global eigenvector – neighbour influence characteristics

(A) Network topology map showing global eigenvector centrality in venous endothelium, where each node (dot) represents an individual endothelial cell, and lines represent edges (connections) between neighbours. Dot plot quantifies global eigenvector measures for cells responding exclusively to acetylcholine (green dots), exclusively to bradykinin (pink dots), or to both stimuli (blue dots, multisensitive), compared to random distributions (grey dots). (B) Dot plots showing the kinetic properties of the Ca^2+^ signal (rise time, fall time and full duration at half maximal [FDHM}) in a target cell with increasing number of active neighbours for all events (10 minute recording). Random data (grey dots) generated by shuffling event times while maintaining a cells total number of events and neighbour relationships. Data represent n = 8 independent experiments from different animals; *P < 0.05, paired Student's t-test.


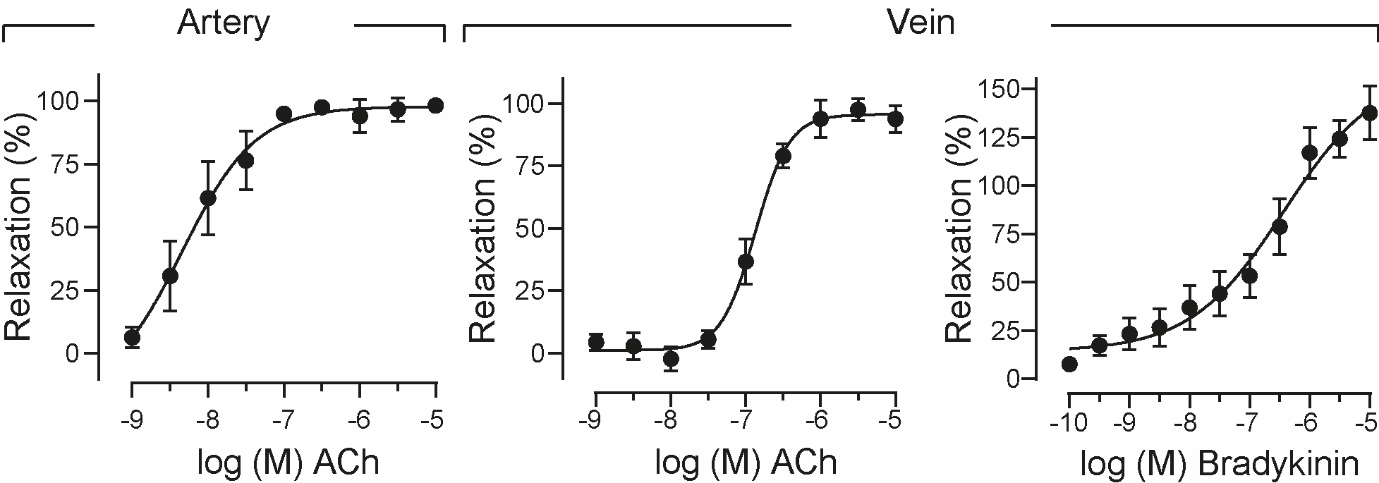


Supplementary Figure 3 - Vasoreactivity of mesenteric arteries and veins to acetylcholine and bradykinin.

Pressurised arteries (70 mmHg) and veins (8 mmHg) were contracted to 80% of baseline with phenylephrine. Cumulative concentration-response curves were generated by adding acetylcholine (ACh) or bradykinin to the superfusate (see methods).
